# Prokaryotic and Highly-Repetitive WD40 Proteins: A Systematic Study

**DOI:** 10.1038/s41598-017-11115-1

**Published:** 2017-09-06

**Authors:** Xue-Jia Hu, Tuan Li, Yang Wang, Yao Xiong, Xian-Hui Wu, De-Lin Zhang, Zhi-Qiang Ye, Yun-Dong Wu

**Affiliations:** 1 Lab of Computational Chemistry and Drug Design, Laboratory of Chemical Genomics, Peking University Shenzhen Graduate School, Shenzhen, 518055 P.R. China; 2 College of Chemistry, Peking University, Beijing, 100871 P.R. China

## Abstract

As an ancient protein family, the WD40 repeat proteins often play essential roles in fundamental cellular processes in eukaryotes. Although investigations of eukaryotic WD40 proteins have been frequently reported, prokaryotic ones remain largely uncharacterized. In this paper, we report a systematic analysis of prokaryotic WD40 proteins and detailed comparisons with eukaryotic ones. About 4,000 prokaryotic WD40 proteins have been identified, accounting for 6.5% of all WD40s. While their abundances are less than 0.1% in most prokaryotes, they are enriched in certain species from Cyanobacteria and Planctomycetes, and participate in various functions such as prokaryotic signal transduction and nutrient synthesis. Comparisons show that a higher proportion of prokaryotic WD40s tend to contain multiple WD40 domains and a large number of hydrogen bond networks. The observation that prokaryotic WD40 proteins tend to show high internal sequence identity suggests that a substantial proportion of them (~20%) should be formed by recent or young repeat duplication events. Further studies demonstrate that the very young WD40 proteins, *i*.*e*., Highly-Repetitive WD40s, should be of higher stability. Our results have presented a catalogue of prokaryotic WD40 proteins, and have shed light on their evolutionary origins.

## Introduction

WD40 domain-containing proteins, constituting one of the most abundant protein families in eukaryotes, often serve as scaffolds in assembling functional complexes by interacting with other macromolecules^1^. They play essential roles in numerous eukaryote-specific cellular processes such as G-protein-mediated signal transduction^2^, transcription regulation^3^, ubiquitin-dependent protein degradation^4, 5^, and chromatin modification^6^.

A typical WD40 domain is a propeller-like structure consisting of 7 repeating blades, each of which contains 40–60 residues and folds into four anti-parallel β-strands (Supplementary Fig. S1a)^7^. The conserved tryptophan-aspartic acid (WD) motif and the length of 40 are adopted to name the repeats and the domains conventionally. There usually exists a shift of one β-strand between the repetitive blades in the structure and the repeating units in the sequence, resulting in a “Velcro” that can close the β-propeller (Supplementary Fig. S1a,b)^8^. While sequences of the repeats are very diverse, their structures remain quite similar^9, 10^. WD40 blades are featured with two prevalent structure motifs, one of which is the stability-contributing side chain hydrogen bond network, formed by residues of aspartic acid, histidine, serine (or threonine), and tryptophan (DH[S/T]W, or tetrad)^11, 12^. Another is the β-bulge, which in combination with the tetrad creates a strategy for prediction of hotspot residues and potential interactions^13^.

In contrast to their high abundance in eukaryotes, WD40 proteins were considered rare in prokaryotes^7^, and it was not until 1996 that the first prokaryotic WD40 protein, PkwA, was discovered^14^. Subsequently, several other prokaryotic WD40 proteins in bacteria have been further identified and studied^15–17^. However, comprehensive identification and characterization, including the abundance, the taxonomic distribution, the distinct features, and the putative functions, of WD40 proteins in Prokaryota are so far unexplored.

The widely accepted model of the formation of propellers (including WD40 domains) hypothesizes that the intra-gene duplications of ancestral peptide genes created these globular domains, which was then followed by sequence diversification and family expansion^18, 19^. These duplication events are believed to have occurred repeatedly in the evolutionary history, and may still continue^20^. Accordingly, the current repertoire of WD40 domains is expected to contain both ancient and young ones, and the sequence identity between repeats within the same domain can be adopted to roughly measure the time that has passed since the intra-gene duplication event happened. Nevertheless, this correlation between within-domain sequence identity and evolutionary age requires confirmation at the DNA level, as the high sequence identity may also be caused in ancient WD40s with purifying selection acting on them. In addition, further depiction of the abundance of young WD40 domains and their taxonomic preference remains largely undetermined.

To cope with these questions, systematic investigations of prokaryotic WD40 proteins and comparison with eukaryotic proteins are necessary, and the rapid accumulation of genomic and proteomic sequences in recent years and the availability of WD40 repeat protein structure prediction program (WDSP)^21, 22^ make these tasks feasible. The identified WD40 proteins and the annotations from WDSP, including predicted secondary structures, putative tetrad hydrogen bond networks, and potential protein-protein interaction hotspots, provide numerous materials to perform these analyses.

In this work, we first systematically identified and annotated all WD40 proteins from the UniProtKB^23^. Then their abundances in prokaryotes and eukaryotes were compared. Additionally, we illustrated their detailed taxonomic distribution in bacteria, and predicted the potential functions of some WD40s using gene neighbourhood analysis. The repeat numbers, the tetrad hydrogen bond network numbers, and the repeat sequence identities of WD40s were also compared between prokaryotes and eukaryotes. Moreover, we defined a subgroup of WD40 proteins with high internal sequence identity, termed Highly-Repetitive (HR), and studied their evolutionary implications and their specialized characteristics of stability.

## Results

### Identification of ~4,000 prokaryotic WD40 proteins

**Figure 1:**
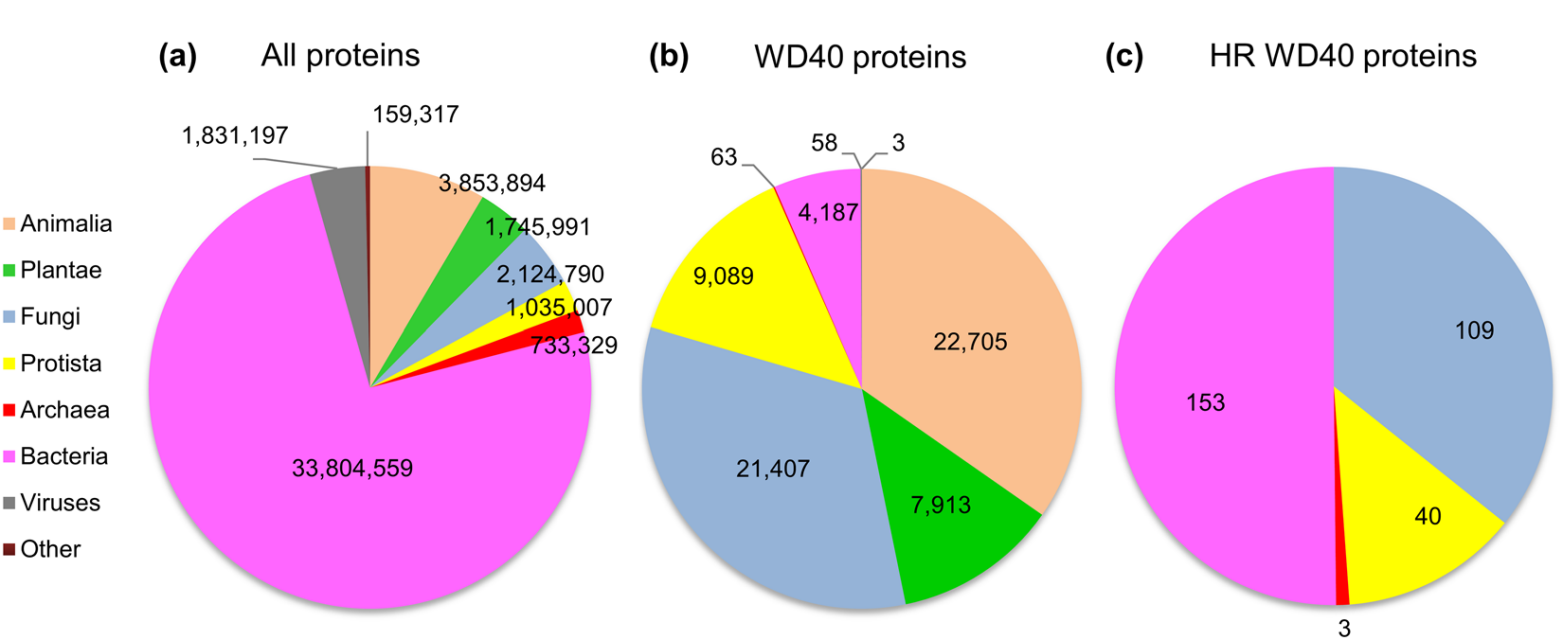
The number of proteins in different taxonomic categories. Different taxonomic categories are shown in different colours. (**a**) All proteins from UniProtKB, (**b**) WD40 proteins, (**c**) HR WD40 proteins.

According to the pipeline described in the Methods section (Supplementary Fig. S2), we curated 65,425 WD40 proteins, including their repeat boundaries, predicted secondary structures, and hydrogen bond networks. Statistics show that most of the proteins (76.26%) in the UniProtKB^23^ dataset come from prokaryotes, while only a small part (19.34%) have eukaryotic sources (Fig. 1a). Nevertheless, 93.41% of the WD40 proteins belong to eukaryotes (Fig. 1b). These results are consistent with the previous consensus that WD40 proteins exist mainly in eukaryotes^7, 24^.

WD40 proteins had been considered rare in prokaryotes^7^, but we identified a considerable number of prokaryotic WD40 proteins. In this work, with a comprehensive set of protein sequences as input and a sensitive prediction method, we identified 4,187 bacteria and 63 archaea WD40 proteins, accounting for 6.50% of the total (Fig. 1b and Supplementary Dataset).

**Figure 2:**
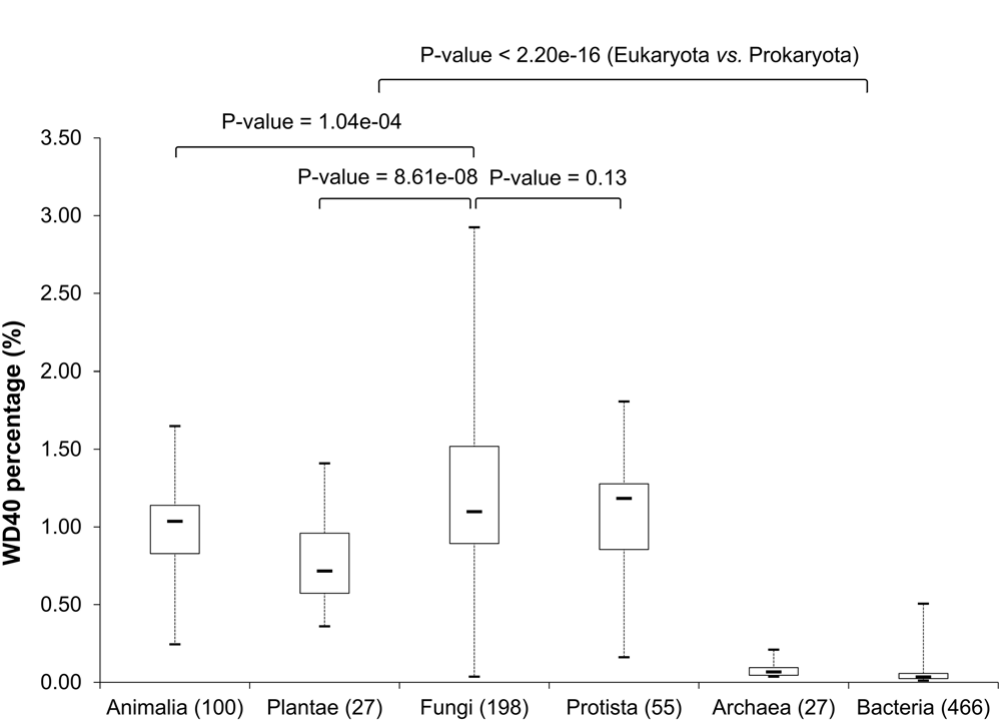
Boxplot of WD40 protein abundances in different taxonomic categories. The vertical axis denotes the percentage of WD40 proteins in the complete proteome of an organism. Each taxonomic category corresponds to a boxplot, showing the maximum, 75% quantile, median, 25% quantile, and the minimum of the WD40 abundance. The numbers in the parentheses give out the amount of organisms in each category. The *p*-values derived from Wilcoxon rank sum tests are annotated at the top.

We compared the abundances of WD40 proteins in different taxonomic groups by calculating the percentages of WD40 proteins in each complete proteome (Table 1). First, WD40 proteins can be found in all eukaryotic proteomes, but they are found in only a small part of prokaryotic proteomes: twenty-seven of 134 (20.15%) archaea proteomes and 466 of 1,679 (27.75%) bacterial proteomes contain WD40 proteins. Second, the percentages of WD40 proteins in eukaryotic proteomes are significantly higher than in prokaryotic proteomes (*p*-value < 2.20e-16, fold change ~17.10, statistical details described in Methods) (Table 1 and Fig. 2). The average is greater than 0.7% in each taxonomic category of the eukaryotes, but it is less than 0.1% in both taxa of the prokaryotes. Third, within eukaryotes, the abundances of WD40 proteins in Fungi are higher than in Animalia and Plantae. The fold changes of the WD40 percentages are 1.23 and 1.57 for the comparison of Fungi to Animalia and Plantae, respectively (*p*-values = 1.04e-04 and 8.61e-08, respectively, statistical details described in Methods) (Table 1 and Fig. 2). Fourth, the percentages of WD40 proteins in Fungi proteomes vary widely, from 0.04% to 2.93%. This phenomenon might be due to a particularity of the taxonomy of fungi: species in fungi are highly diverse, and cover a broad spectrum of organisms, from unicellular to multicellular, with large differences between them^25^. These results furthered our understanding about the abundances of WD40 proteins in different taxonomic groups and their differences.

**Table 1:** The abundances of WD40 proteins in different taxonomic categories.

Category	# of complete proteomes	# of proteomes containing WD40s	% of proteomes containing WD40s	Average % of WD40s per proteome (abundance)
Animalia	100	100	100	0.98
Plantae	27	27	100	0.77
Fungi	198	198	100	1.21
Protista	55	55	100	1.08
Archaea	134	27	20.15	0.08
Bacteria	1679	466	27.75	0.06

### Prokaryotic WD40 proteins are enriched in Cyanobacteria and Planctomycetes

As only a small proportion of prokaryotic genomes encode WD40 proteins, it is of great interest to explore from which groups of species the WD40 proteins derive. In this study, we focused on bacteria, the majority of prokaryotes.

**Figure 3:**
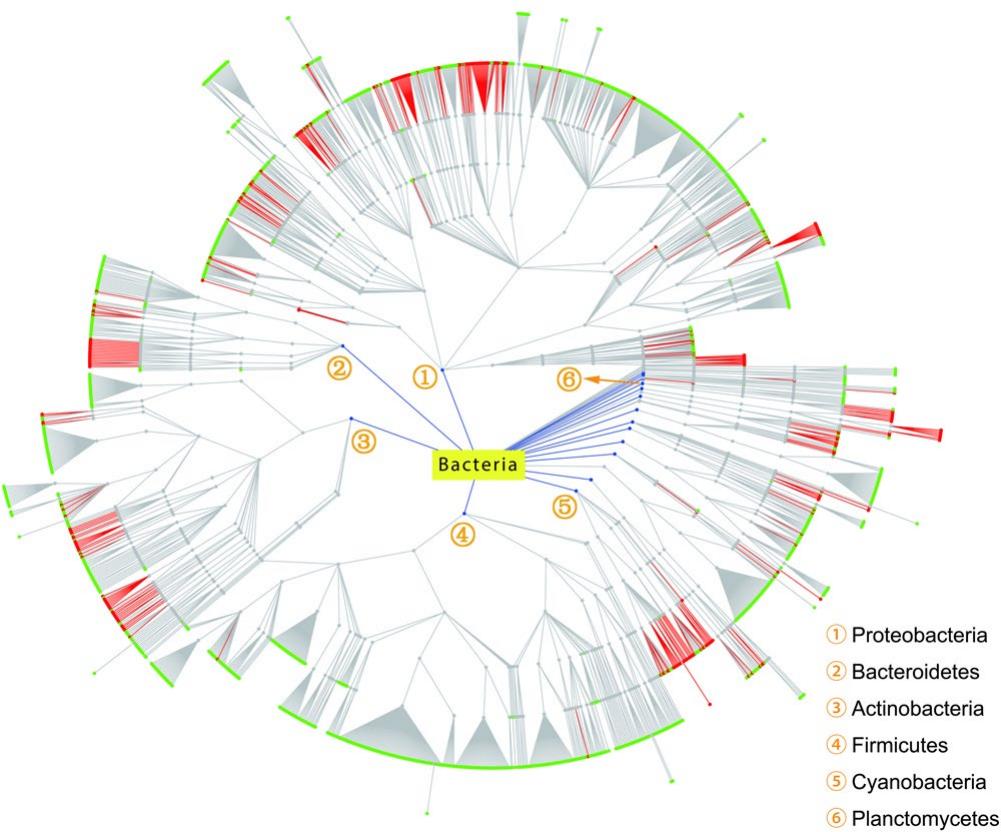
Taxonomic distribution of bacterial WD40 proteins. Near the centre, blue lines and dots represent the phyla that contain organisms possessing WD40 proteins, and the names of several major phyla are shown. Near the periphery, red lines and dots denote the organisms possessing WD40 proteins, while green dots show the organisms not possessing any WD40 proteins.

Our dataset contains 1,679 bacterial species with complete proteomes, and we were able to identify 1,337 WD40 proteins from 466 of them (Supplementary Table S1). After ranking them by the abundance of WD40 proteins, 8 of the top 10 organisms belong to the phylum of Cyanobacteria and the other 2 organisms are from Planctomycetes and Actinobacteria (Supplementary Table S2). To further visualize the distribution of WD40 proteins, we plotted a taxonomic tree for the 1,679 bacterial species (Fig. 3). At the top level, there are a total of 30 phyla, 20 of which contain bacterial organisms possessing WD40 proteins (Fig. 3 and Supplementary Table S1).

On one hand, certain phyla contain organisms with high abundances of WD40 proteins. Specifically, each organism in Planctomycetes and Cyanobacteria, contains on average 13 and 10 WD40 proteins respectively (Supplementary Table S1). On the other hand, though WD40 proteins exist widely in many organisms of Actinobacteria and Proteobacteria (containing 248 and 413 WD40 proteins, respectively), the average number per proteome is very low (4 and 2, respectively). Firmicutes, one of the largest bacteria phyla, contains 5 WD40 proteins in total, revealing that most of organisms in this phylum do not encode any WD40 protein.

It seems that bacteria with abundant WD40 proteins tend to possess complicated cellular structures. Cyanobacteria contain two membranes, and they can perform photosynthesis in distinctive folds in the outer membrane, which makes them very different from other bacteria^26^. Planctomycetes are a type of obligate aerobic bacteria. They have membrane-bound internal compartments, including the paryphoplasm (ribosome-free space), pirellulosome (ribosome-containing space), and nucleoid (condensed nucleic acid region, surrounded by a double membrane)^27^. For example, the chromatin of *Gemmata obscuriglobus* (a species of Planctomycete) is encapsulated by a double-layered nuclear membrane, which is similar to the structure of eukaryotic nuclei^28^. These may indicate that the abundance of WD40 proteins might be associated with cellular structure complexity, but further confirmation requires more detailed investigations.

### Potential functions of prokaryotic WD40 proteins

Although WD40 domains are structurally suitable to serve as scaffolds in protein-protein interactions and complex assembling^29^, what pathways or biological processes they participate in requires more in-depth studies. In prokaryotes, we can make functional inferences by performing gene neighbourhood analysis^30, 31^, which is based on the fact that many prokaryotic genes cluster as a functional unit, *i*.*e*., operon. We chose all the 7 species with complete proteomes from Planctomycetes for gene neighbourhood analysis. As for Cyanobacteria, we chose the same number of species by considering both the WD40 abundance and their taxonomic representativeness (Supplementary Table S3).

**Figure 4:**
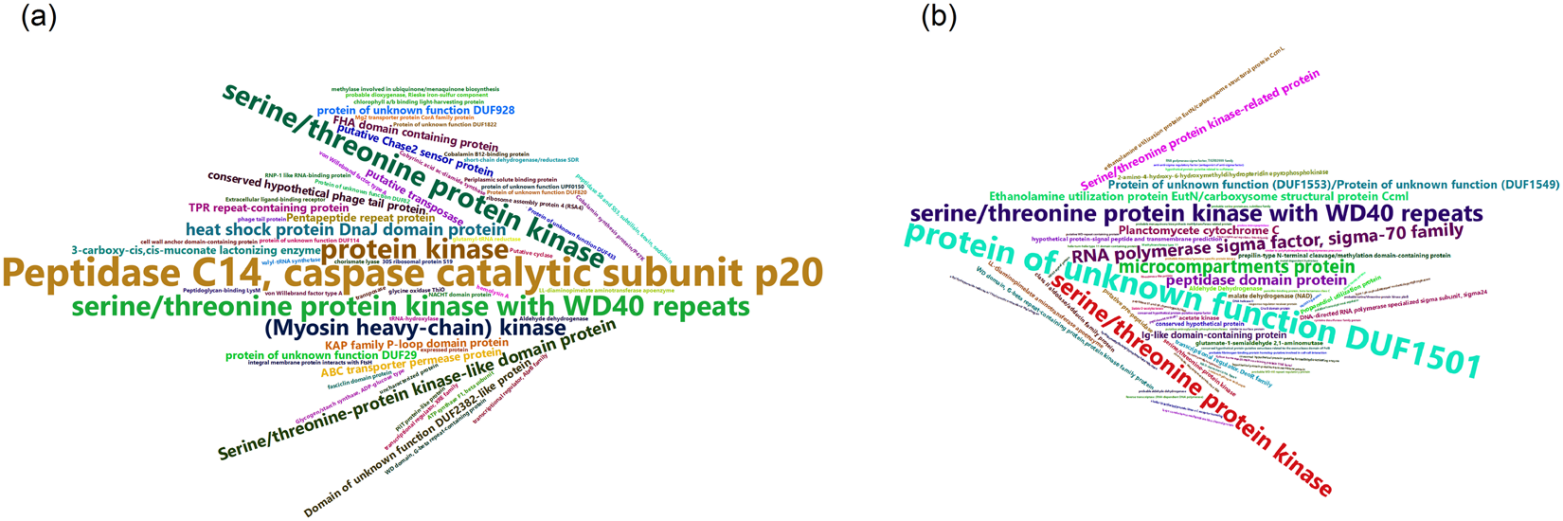
Word clouds for the annotations of WD40s and their neighbouring genes. Annotation phrases are coloured differently, and the sizes represent their relative abundances. (**a**) Cyanobacteria, (**b**) Planctomycetes.

Across these 14 species, the annotations of the WD40 genes and their neighbouring genes were presented as word clouds, showing that the protein products of neighbouring genes of WD40s in Cyanobacteria and Planctomycetes are frequently associated with kinases, especially serine/threonine protein kinase, and that many WD40 genes even encode kinase domains within the same protein (Fig. 4). The association between WD40 and kinase is also observed in eukaryotes, but much less frequently^32^. Specifically, the caspase catalytic subunit p20 of peptidase C14 and the heat shock protein DnaJ domain are often associated with the protein products of the neighbours of WD40 genes in Cyanobacteria (Fig. 4a). The former is a type of enzyme participating in signal transduction in Cyanobaceteria^33^, and its animal homolog can serve as interleukin-1 beta convertase, which plays a role in inflammation through cell communication^34^. The latter is a molecule chaperon component responsible for regulating the ATPase activity of Hsp70^35^. In Planctomycetes, DUF1501 (a type of conserved domain with unknown function) and RNA polymerase sigma-70 factor family are often annotated to the protein products of the neighbours of WD40-coding genes (Fig. 4b). These analyses suggest that many prokaryotic WD40 proteins may function in pervasive catalytic machineries that are involved in signal transduction, protein folding, transcription, *etc*.

**Figure 5:**
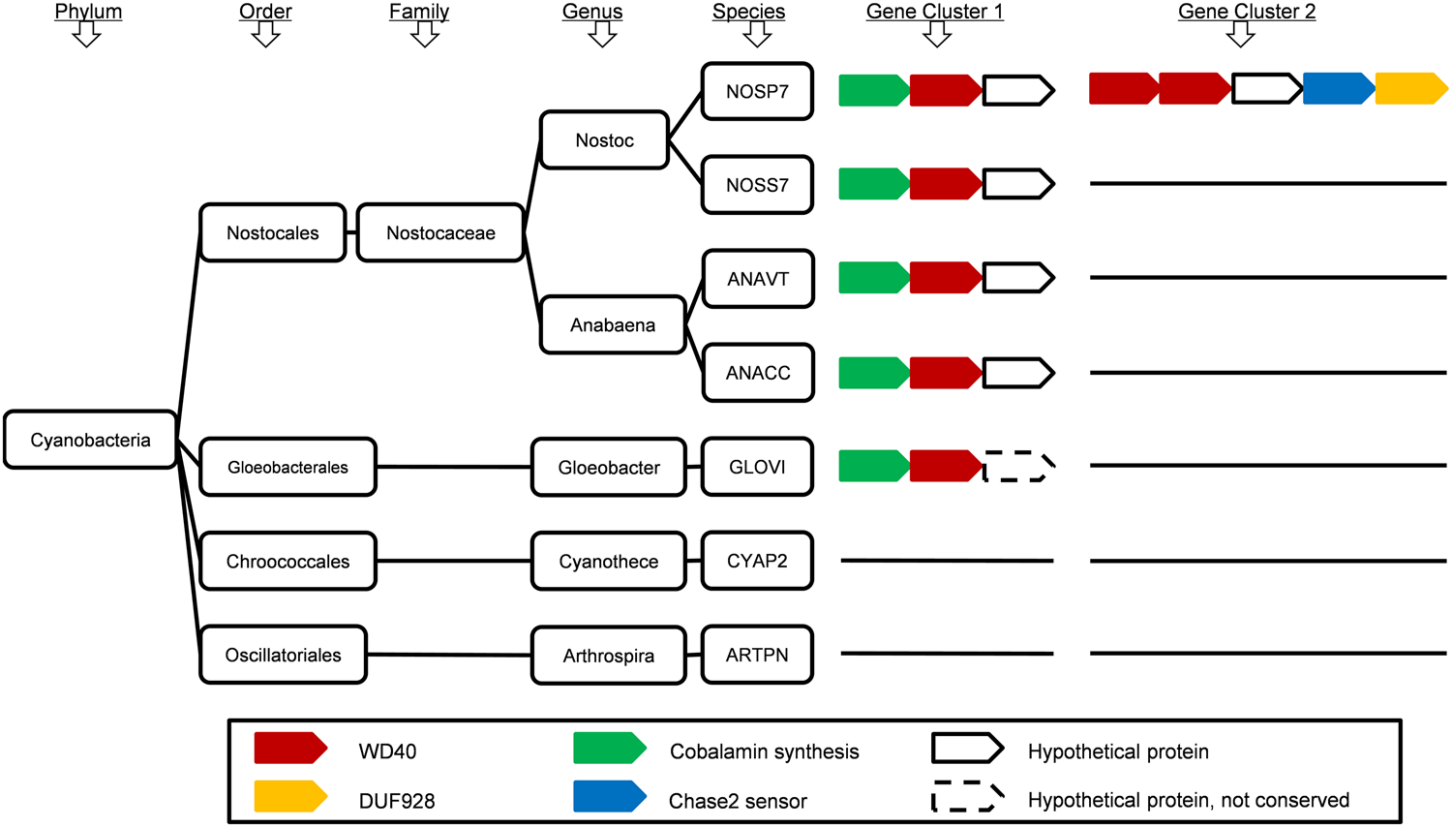
Two examples of gene neighbourhood analysis in Cyanobacteria. The taxonomic relationship of the eight Cyanobacteria species is depicted on the left, and the taxonomic levels are denoted on the top. Two gene clusters identified by gene neighbourhood analysis are on the right. Gene cluster 1 is conserved in the Order of Nostocales, and gene cluster 2 is species-specific. Annotations of these genes are shown on the bottom.

Furthermore, we analysed the conservation of WD40 proteins and their neighbours across different Cyanobacteria species. If a gene cluster is conserved across different species within a taxon, it is of high possibility that these genes may participate in functions important for this entire taxon. In this analysis, we have not found any WD40 with its neighbours broadly conserved across all the 7 selected Cyanobacteria species, but some cases are conserved across species from the same family. For example, the gene encoding WD40 protein B2J567 in NOSP7 has two neighbours, one of which is annotated as encoding “cobalamin synthesis protein, P47K”. This WD40 gene and its neighbours are not only conserved in NOSS7 from the same genus of *Nostoc*, but also conserved in ANAVT and ANACC from the genus of *Anabaena* (Fig. 5 and Supplementary Table S4, Gene Cluster 1). These two genera are from the same family (Nostocaceae) and Order (Nostocales). Hence, we speculate that this gene cluster is conservative in the entire Order of Nostocales. We can also find remote homologs of this WD40 and the cobalamin synthesis protein in GLOVI from another Order (Gloeobacterales). Nevertheless, the sequence identity between the WD40 homologs is only 32%; the homologs of the cobalamin synthesis protein are only aligned in a half of the sequence length; and the other neighbouring gene in GLOVI is not homologous to those in the Order of Nostocales. It is well-known that many Cyanobacteria species can synthesis vitamin B12 or pseudocobalamin, which is essential to the nutrient acquisition of many other organisms^36^. In the Order of Nostocales, the WD40 protein B2J567 and its orthologs may participate in this important biological process. In the Order of Gloeobacterales, the homolog of B2J567 may be involved in this process as well, but in a slightly different way as the conservation of the gene neighbourhood in GLOVI is low.

On the other hand, different gene neighbourhoods in a specific lineage may also indicate lineage-specific functions. For example in the NOSP7, we identified 5 closely arrayed genes encoding 2 WD40 proteins, 1 hypothetical protein, 1 CHASE2 sensor protein, and 1 DUF928 protein (Fig. 5 and Supplementary Table S4, Gene Cluster 2). This type of gene organization is only found in NOSP7 in the 7 surveyed Cyanobacteria species. CHASE2 sensor is an extracellular domain which senses the environmental changes and participates in the signal transduction processes^37^. In a study of *Synechocystis sp*. PCC6803, the protein sll1891 containing a DUF928 domain was found to respond to the environmental acid stress^38^. According to these lines of information, we can infer that these two WD40 proteins may be implicated in the signal transduction process that responds to the extracellular signals in the lineage of NOSP7 specifically.

Taken together, the gene neighbourhood analyses have provided rich information concerning their potential functions of prokaryotic WD40 proteins, although we currently cannot answer why they are enriched in Cyanobacteria and Planctomycetes yet.

### The distributions of repeat and tetrad numbers in prokaryotic and eukaryotic WD40 proteins are different

Typically, a WD40 domain is composed of seven repeating units, but other scenarios exist as well. According to the statistics on crystal structures, a WD40 domain may consist of six, seven, eight, or nine repeats. WD40 proteins with 14 repeats can be regarded as forming two typical WD40 domains. For example, B2J0I0_NOSP7 (Gene symbol: *Npun_R6612*, PDB ID: 2YMU, Zeth, *et al*. unpublished), containing 14 repeats, forms two WD40 domains (Supplementary Fig. S1c). Accordingly, the number of repeats of a WD40 protein can be used to infer the number of WD40 domains.

**Figure 6:**
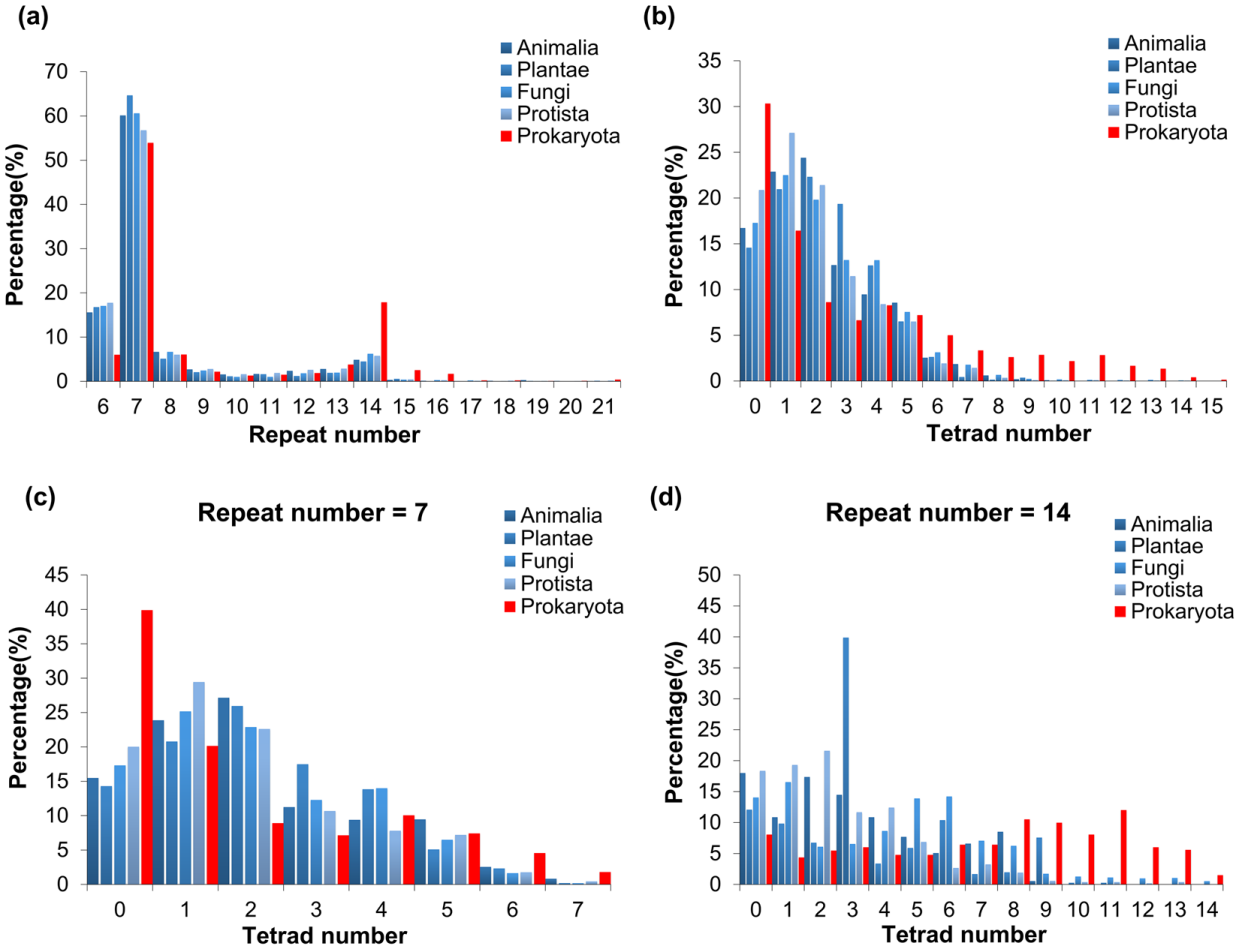
Comparison of the repeat number and the tetrad number distributions between different taxonomic categories. In each panel, the red bars represent the data from Prokaryota, while the blue bars in different colour depth represent data from different eukaryotic categories. (**a**) The distributions of the repeat numbers of WD40 proteins in each taxonomic category. (**b**) The distributions of the tetrad hydrogen bond network numbers of WD40 proteins in each taxonomic category. (**c**) and (**d**) represent the tetrad number distributions of WD40 proteins with 7 repeats and 14 repeats, respectively.

The overall distributions of the repeat numbers are similar between prokaryotes and eukaryotes, but there exists a clear difference. First, most WD40 proteins are composed of seven repeats, which is consistent with the previous inference based on their geometrical, physical, and chemical properties (Fig. 6a)^39^. Second, a considerable amount of WD40 proteins contain six repeats. Since knowledge from crystal structures has shown that some WD40 proteins, such as Sec. 13^40^, are in the form of open propeller with a repeat missing, we can speculate that these WD40 proteins should largely match this scenario. Third, a substantial number of proteins consist of 14 repeats, which can be expected to fold into two WD40 domains (Fig. 6a). The comparison shows that the distributions of different eukaryotic categories are similar, but a much higher percentage of prokaryotic WD40 proteins than that of eukaryotic WD40 proteins contain 14 repeats, suggesting that a higher proportion of WD40 proteins in prokaryotes contain multiple WD40 domains than in eukaryotes. Another observation is that the proportion of prokaryotic WD40 proteins with 6 repeats is only about one third of those of eukaryotic WD40s with 6 repeats, but a reasonable interpretation of this requires further investigation.

The tetrad hydrogen bond network, contributing to the thermal stability of the domain^11, 12^, is considered to be a unique feature of WD40 proteins, so we compared the distributions of this feature between prokaryotes and eukaryotes as well (Fig. 6b). Like the repeat numbers, the tetrad numbers distribute similarly among different eukaryotic kingdoms. However, compared to those in eukaryotes, WD40 proteins in prokaryotes are obviously inclined to enrichment at both ends. Enrichment at the right end indicates that a larger proportion of prokaryotic WD40 proteins, than that of eukaryotic ones, possess high level of tetrads and may thus possess high thermal stability. The left end is another extreme, *i*.*e*., the proportion of WD40 proteins without any tetrad in prokaryotes is also higher than in eukaryotes.

Since one repeat can possess no more than a single tetrad, there may exist a correlation between the distributions of tetrad number and repeat number. In order to remove the effect of repeat numbers on the tetrad number distribution, we further plotted the tetrad distributions for WD40 proteins with 7 and 14 repeats, *i*.*e*., one domain or two domains, respectively (Fig. 6c,d). For WD40 proteins with one domain, the tetrad distributions are similar to the overall distributions (Fig. 6b). For WD40 proteins with two domains, the prokaryotic proteins evidently contain more tetrads than eukaryotic ones, which still supports the speculation that a larger proportion of prokaryotic WD40 proteins are of high thermal stability. The bimodal distribution of tetrad numbers in prokaryotes also shows that the prokaryotic WD40 proteins can be divided into different groups with respect to their tetrad abundance.

### Prokaryotic WD40 proteins show higher internal sequence identity than eukaryotic ones

In order to inspect the characteristics of prokaryotic WD40 protein sequences, we constructed the sequence logos of the WD40 repeats from prokaryotic and eukaryotic WD40 proteins (Supplementary Fig. S3a,b, see the Methods section for the naming conventions of strands and loops). As expected, the sequence logos of WD40 repeats in prokaryotes and eukaryotes are similar overall. The most frequently encountered residues at each site are almost the same, but several positions are more conserved in prokaryotes. The most evident one is the “Ser-Pro-Asp-Gly”, the SPDG motif on L_ab_, which is a motif frequently found in WD40 proteins^20^. In addition, tryptophan (W) at the S_c_ (position 37) is also evidently more conserved in prokaryotes. Although we have observed differences, the sequence logos only provide a coarse impression about the conservation, and more detailed mining is described as follows.

**Figure 7:**
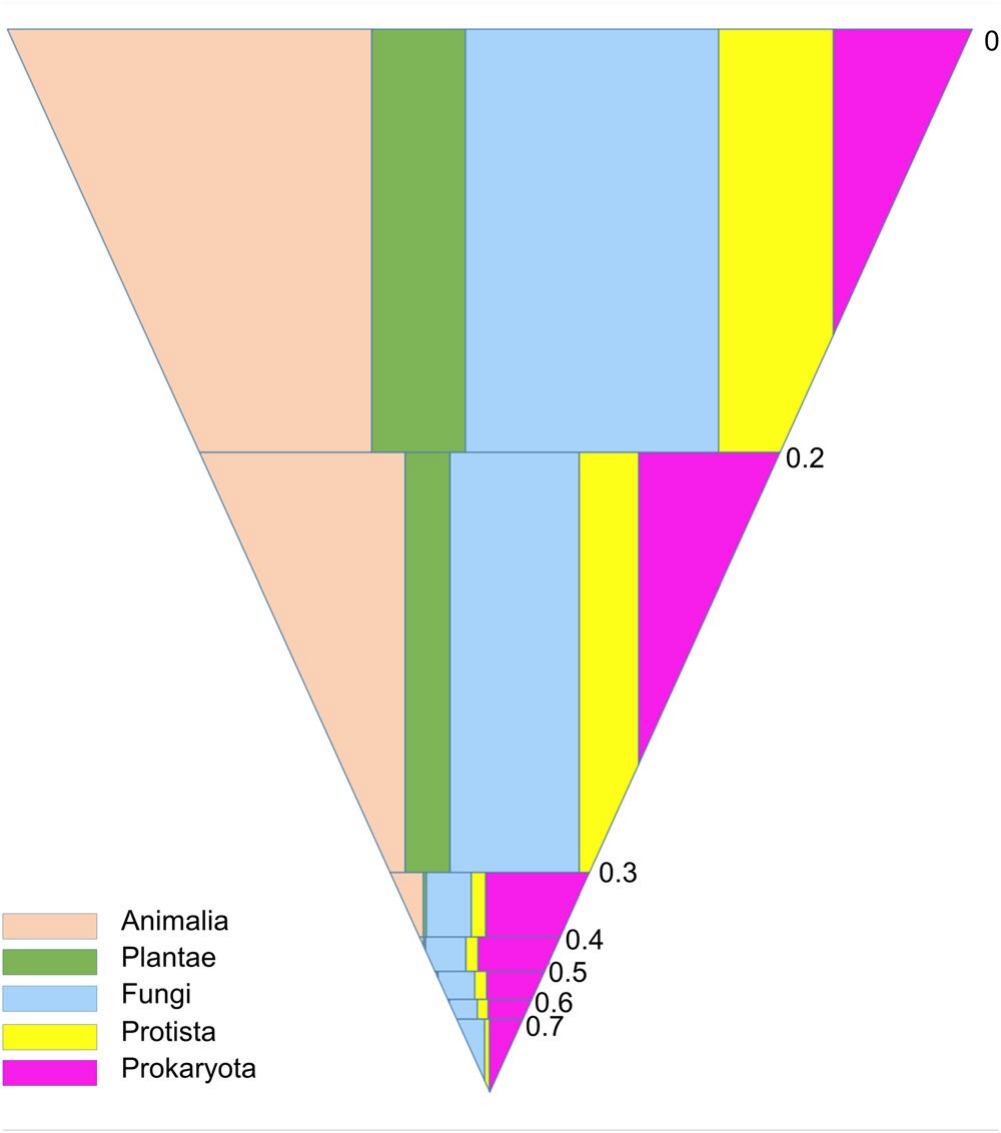
Relative amounts of WD40 proteins at different levels of internal sequence identity in different taxonomic categories. Different colours correspond to different taxonomic categories, as denoted at the bottom on the left. The horizontal layers correspond to different levels of internal sequence identity, whose values are marked on the right. The sizes of the areas represent the relative amounts of WD40 proteins matching the corresponding internal sequence identity level and taxonomic category.

As mentioned in the Introduction section, the sequence identities between repeat units within the same domain or protein can provide clues regarding their evolutionary insights, so it is of great interest to explore them systematically. For each WD40 protein, we calculated the internal sequence identity level in the way described in the Methods section, and the proteins in different taxonomic categories were grouped accordingly (Fig. 7 and Supplementary Table S5). It is clear that almost all WD40 proteins in high eukaryotic organisms (Animalia and Plantae) have an internal sequence identity of less than 0.4, while those in Prokaryota are distributed in a wide spectrum and range up to 0.9. On one hand, about 20% of the prokaryotic WD40 proteins have an internal sequence identity greater than 0.4. On the other hand, of the WD40 proteins with internal sequence identity greater than 0.4, more than half are from prokaryotes, especially bacteria, though bacterial WD40 proteins account for only 6.40% of the total. Interestingly, although Fungi and Protista are eukaryotic, some 2% of their WD40 proteins have an internal sequence identity greater than 0.4. The distribution patterns in Animalia and Plantae are consistent with a previous understanding that the repeat sequences of WD40 proteins are of high diversity. After focusing on the repeat comparison within the same protein, our analysis revealed that a considerable number of WD40 proteins from prokaryotes and lower eukaryotes (Fungi and Protista) demonstrate high internal sequence identity. For the convenience in the following analyses, we define the Highly-Repetitive (HR) and Moderately-Repetitive (MR) WD40 proteins by using 0.7 and 0.4 as the cutoff of internal sequence identity (details in Methods).

### Higher internal sequence identity indicates more recent evolutionary origin

It is well accepted that the propeller domains were formed by duplication events that have acted on ancestral repeat-encoding peptide genes (formation by repeat duplication)^20^. In the process of evolution, with the accumulation of mutations, the protein sequences of the repeats from the same domain became diversified, and new functions were able to emerge gradually^18, 20^. Hence, the level of internal sequence identity is correlated with the evolutionary time of the repeat duplication events: the higher the internal sequence identity is, the more recent the formation of the domain is. However, it is also possible that the domain was formed by an ancient repeat duplication event, but the internal sequence identity remains high due to purifying selection acting on the protein sequence. In order to figure out which scenario is prevalent, we analyzed the synonymous substitution rate (*d*S) between repeats within the same protein, as the *d*S values are largely neutral and thus could serve as a proxy of the divergent time^41, 42^.

**Figure 8:**
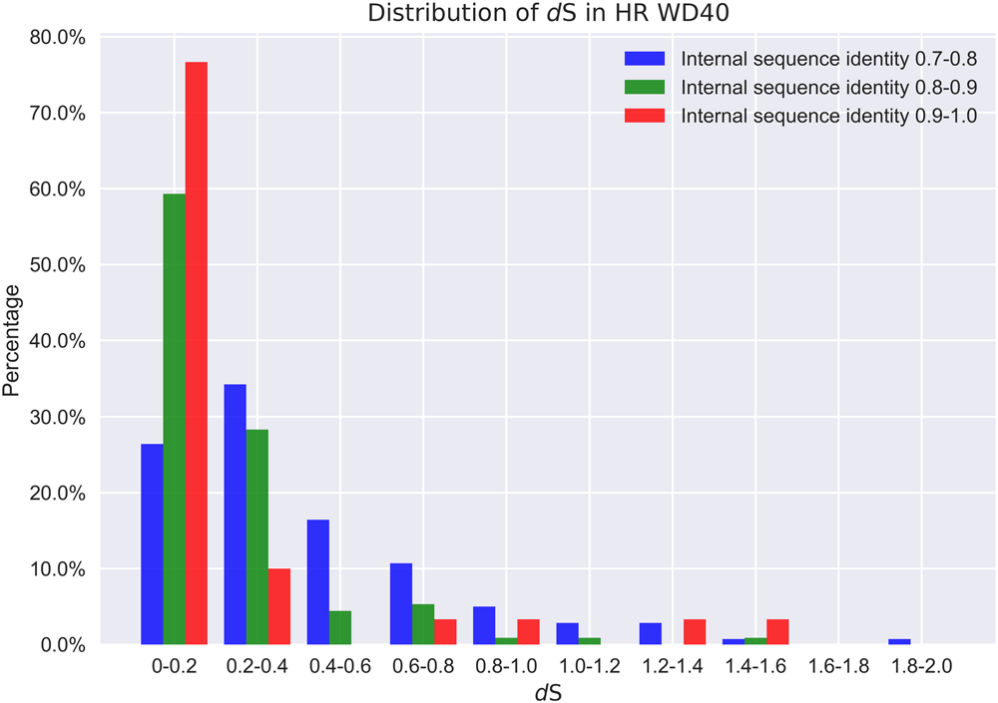
The distributions of *d*S in HR WD40 genes at different internal sequence identity levels. For each HR WD40 gene, *d*S values are calculated between all the repeat pairs, and the median is chosen for the analysis. The distributions are plotted for HR WD40 genes at the internal sequence identity levels of 0.7–0.8, 0.8–0.9, and 0.9–1.0, respectively.

We have calculated the *d*S values for the HR WD40 proteins with internal identity between 0.7–0.8, 0.8–0.9, and 0.9–1.0, respectively. For each protein, a median *d*S value was derived according to the details in the Methods. In the distributions plot, *d*S values tend to be small (often less than 0.4, Fig. 8). This apparently indicates that the majority of these WD40 proteins should have originated from recent internal duplication events. In addition, the plot shows that the higher level the internal identity is, the distribution of *d*S is more enriched near 0. That is, *d*S increases with the decrease of internal sequence identity at protein level. Actually, for the identity group 0.9–1.0, nearly 90% of proteins show a *dS* less than 0.4, while this proportion drops to ~60% for the group 0.7–0.8. Overall, since a small *d*S value means a short evolutionary time, this observation confirms the argument that higher internal sequence identity implies more recent evolutionary origin in most cases.

On the other hand, we do have observed that certain HR WD40s are of ancient origin, *i*.*e*., with high *d*S values (Fig. 8). We further calculated the ratios of non-synonymous substitution rate to synonymous substitution rate (*d*N/*d*S or ω) for proteins with *d*S no less than 1.0 (but less than 2.0 since larger *d*S values cannot be reliably estimated), and have found that most of these ω values are far less than 1.0 (median of ω: 0.19). As small ω values indicate high purifying selection pressure, this result reveals that these ancient HR WD40s should have been kept at high internal sequence identity level by the purifying selection. Although this type of cases exist, their amount is very small (Fig. 8), and will not affect the overall correlation between the internal identity and the evolutionary time of repeat duplication events.

Based on the above analyses, we can infer the relative amount of ancient and young WD40 proteins in nature according to the internal sequence identity distribution, though we may overestimate the amount of young WD40 proteins. If setting 0.4 as the cutoff of internal identity between ancient and young, we can observe ~20% of prokaryotic and ~2% of lower eukaryotic WD40 proteins, but not several sporadic proteins, are evolutionarily younger than others, although the ancient WD40s still constitute the majority.

Previous studies on human WD40 proteins have revealed that domain-level duplications are prevalent in eukaryotes, where they serve as an important mechanism for the expansion of the WD40 and many other protein families^43, 44^. In this work, we have found that repeat-level duplications, which were responsible for the domain formation and were previously assumed to happen mainly in evolutionarily ancient stages, also frequently occurred recently in prokaryotes and lower eukaryotes. It seems that lower organisms until today, have continued to use the repeat-level duplication frequently while higher organisms have almost abandoned it.

### Highly-Repetitive WD40 proteins

Considering that there are substantial WD40 proteins with recent evolutionary origin in lower organisms including prokaryotes, protists, and fungi, it is promising to analyse their sequence and structure features specifically. For this purpose, we specifically analysed the 305 HR WD40 proteins, most of which should be very young according to the analyses above. Of them, 50.16% and 35.74% are from bacteria and fungi, respectively, and none comes from animals or plants (Fig. 7 and Supplementary Table S5, and Supplementary Dataset).

Overall, the HR WD40 repeats are much more conserved than general WD40 repeats (Supplementary Fig. S3c, and an example in Supplementary Fig. S4). Notably, although most sites of HR WD40 repeats show high conservation, some are of high diversity, such as the potential interaction hotspot sites^13^ (red stars in the alignment positions). This observation suggests that the members of HR WD40 proteins may have evolved with specific hotspot residues to adapt different interaction partners, though they have not evolved as long as other WD40 proteins.

**Figure 9:**
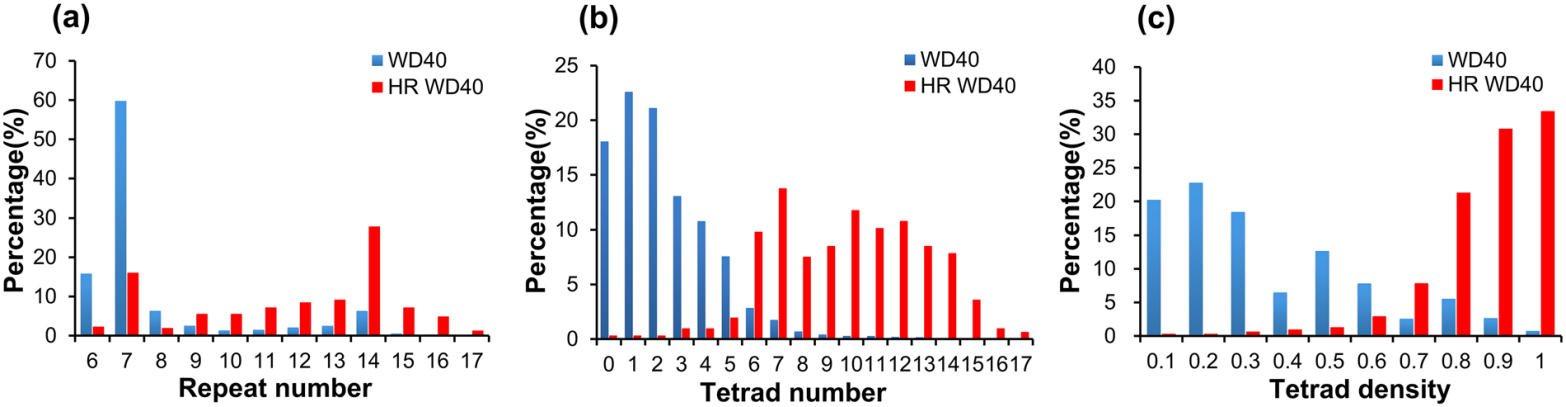
Comparison of the repeat number and the tetrad number distributions between HR WD40 and all WD40 proteins. In each panel, the red bars represent the data from HR WD40 proteins, while the blue bars denote the data from all WD40 proteins. (**a**) The distributions of the repeat numbers. (**b**) The distributions of the tetrad hydrogen bond networks numbers. (**c**) The distributions of tetrad densities (measured by the ratio of the tetrad number to the repeat number).

Based on their distribution plots, HR WD40 proteins tend to contain more repeats and more tetrad hydrogen bond networks than others (Fig. 9a,b). As mentioned above, a large repeat number implies that the protein may be composed of more than one domain and a large tetrad number indicates that the protein may be more stable. On one hand, if a protein contains multiple WD40 domains, it may present more interaction interfaces, and thus serve as a larger scaffold for complex assembly. This may suggest that a large number of HR WD40 proteins are prone to form bulkier complexes. On the other hand, the observation that the HR WD40 proteins have a large tetrad number indicates that the majority of HR WD40 proteins may be of higher thermal stability than other proteins. Nevertheless, the high level of tetrad number may be correlated with the high level of repeat number as mentioned. In order to eliminate this effect, we further calculated the ratio of tetrad number to repeat number, namely tetrad density, for each protein, and compared their distributions between HR and general WD40 proteins (Fig. 9c). It remains clear that the tetrad density is still much higher in HR WD40 proteins than in general WD40s, and this confirms our speculation that most HR WD40s have higher thermal stability.

From another point of view, we can say that WD40 domains were very stable at the moment of their formation by repeat duplication. Previous studies have raised the opinion that the stability of a protein promotes its ability to evolve in many cases, which relates biophysics to molecular evolution^45, 46^. The rationale behind is that the extra stability of a protein can tolerate destabilizing mutations that may lead to new functions, *i*.*e*., there is a “trade-off” between the function and the stability. If this rule is applicable to HR WD40s, their high stability may indicate that they might have large evolvability. Hence, the HR WD40 proteins can serve as materials for further investigation of this argument. Another aspect is that the high thermal stability of HR WD40 proteins will also offer promise for engineering of heat-tolerant molecules with designed functions.

## Discussion

Repeating sequences are frequently found in non-coding regions as well as in protein-coding regions, especially in eukaryotic genomes^47^. As they undergo relaxation of the constraints of sequence conservation due to their binding functions ensuing from multiple repeats, they are thought to evolve faster than general sequences^18^. This feature makes internal repetition a “cheap” approach to obtaining complicated functions in the evolution of eukaryotes, and may explain why eukaryotic repeats are much more prevalent than prokaryotic repeats^47^. WD40 repeats apparently conform to this scenario.

Although the number of prokaryotic WD40 proteins is much less than that of eukaryotic ones, we have identified about 4,000 prokaryotic WD40 proteins from the UniProtKB database^23^. They are particularly common in some species from Cyanobacteria and Planctomycetes. Many Cyanobacteria species contain up to 20 WD40 proteins, suggesting that WD40 proteins are not rare in certain prokaryotes. It seems that bacteria with complicated cellular structures tend to possess more WD40 proteins, which suggests that functional associations between them might exist. By using gene neighbourhood analysis, we predict that many prokaryotic WD40 proteins may be frequently involved in machineries of signal transduction, protein folding, transcription, enzyme catalysis, nutrient synthesis, *etc*. However, the reason why prokaryotic WD40s are enriched in Cyanobacteria and Planctomyctes remains unclear.

We have shown that the tetrad numbers of prokaryotic WD40 proteins are distributed in a bimodal pattern, suggesting that WD40 proteins in prokaryotes can be divided into different groups (Fig. 6b,c). The subsequent exploration of internal sequence identity also presents evident patterns of separation: prokaryotes contain a substantial proportion of WD40 proteins with high internal identity (Fig. 7). We further observed that HR WD40 proteins contain a highly dense hydrogen bond network (Fig. 9c), suggesting that it may be the high proportion of HR WD40 proteins in prokaryotes that results in the bimodal distribution of tetrads.

As mentioned in the Introduction section, the hypothesis of the evolution of the propeller domain (including the WD40 domain) assumes that they were formed by intra-gene duplication of ancestral peptide genes, and the level of internal sequence identity of repeats indicates the evolutionary time of these duplication events^18–20^. By using *d*S values calculated from the DNA sequences of the repeats within the same protein, we have confirmed the validity of this correlation. Our results further show that substantial WD40 domains have been formed from the duplication events of repeats more recently than others, and the very young WD40 proteins (HR WD40) are mainly derived from prokaryotes and lower eukaryotes. The high stability and the recent evolutionary origin of HR WD40s suggest they may be utilized to explore the possible link between the stability and evolvability^48^. The domain-level duplication and the repeat-level duplication, as two mechanisms of WD40 family expansion, remind us that molecular evolution may be modular at different scales.

Where the prokaryotic WD40 repeats have come is currently inconclusive. In a book chapter by Smith T.F.^24^, the author has discussed several possibilities, and prefers to the hypothesis that WD40-repeat proteins first originated in the ancestor of eukaryotes, and that horizontal gene transfer (HGT) events have given birth to the prokaryotic WD40s. This speculation is based on the fact that WD40 proteins widely participate in fundamental eukaryotic cellular processes such as eukaryotic signal transduction, RNA processing, cytoskeletal assembly, and transcription initiation, all of which have been considered as components of the last eukaryotic common ancestor (LECA)^24^. We further analysed the LECA proposed by Koonin E.V.^48^, and found that all the 96 LECA WD40 proteins can hit at least one prokaryotic WD40 protein by using BLASTP^49^ at the E-value of 1E-4 (Supplementary Methods). This homology suggests that prokaryotic and eukaryotic WD40s may have inherited from the last universal common ancestor (LUCA) or may have originated within one taxonomic domain but have spread to other taxonomic domains by HGT. On the other hand, a recent inference of the last universal common ancestor (LUCA) contains 355 genes^50^, none of which can be annotated as WD40 (Supplementary Methods). This denies the argument of prokaryotic WD40s’ origin from LUCA, and leaves the HGT speculation seemingly probable.

However, evidences supporting the opposite also exist. Different criteria can lead to different inference of LUCA. According to the annotations of eggNOG^51^, nearly one half of the 65425 WD40s identified in this work can be traced back to LUCA (Supplementary Methods), suggesting that prokaryotic WD40s might have been inherited from it. As for the possibility of HGT from eukaryotes to prokaryotes, the pathogenic prokaryotes from the Phylum of Firmicutes, which are often closely related to higher eukaryotes by parasitism or symbiosis, contain only 5 WD40 proteins in 5 of 364 species according to our dataset. We further predicted the potential horizontal transferred genes (HTG) for these 5 bacteria by using HGT-Finder^52^, and found that these 5 WD40 proteins are not likely to be horizontally transferred from eukaryotes (Supplementary Methods). These data showed other possibilities of the origin of prokaryotic WD40s, leaving it controversial yet.

At the repeat level, we cannot infer the exact origin of the first prokaryotic WD40 repeat based on the discussion above. However, at the domain or protein level, our analyses of HR WD40s can shed light on the domain formation from repeats. Taking prokaryotic B2J0I0_NOSP7 as an example (Supplementary Fig. S4), we found that its internal sequence identity is greater than 0.9, and the median *d*S between repeats is 0.0, indicating that it may have been formed through extremely recent repeat duplication events. Therefore, we infer that this process of domain formation should have happened in NOSP7, but not in eukaryotes followed by HGT to NOSP7. After running an online BLAST^49^ search against the eukaryotic proteins, we found the best hit with sequence identities of 66% (B8LXS9_TALSN from *Talaromyces stipitatus*, a kind of fungi), far less than the internal sequence identity, which confirmed our speculation. That is, regardless of what sources the repeats come from, the process of formation of certain prokaryotic WD40 domains from repeat duplications may happen within prokaryotes themselves.

## Conclusion

In summary, we systematically identified all putative WD40 proteins from the UniprotKB database, and annotated their secondary structures and tetrad hydrogen bond networks. Among them, there are about 4,000 prokaryotic WD40 proteins, accounting for 6.5% of the total. About 20%~30% of the prokaryotic genomes encode WD40 proteins, but their abundances are generally less than 0.1%. They are mainly enriched in Cyanobacteria and Planctomycetes, where they may participate in various machineries responsible for prokaryotic signal transduction, protein folding, transcription, enzyme catalysis, nutrient synthesis, *etc*. Prokaryotic WD40 proteins tend to contain more WD40 domains than eukaryotic ones, and that the distribution of tetrad counts of prokaryotic WD40 proteins is more enriched at the two ends, *i*.*e*., no tetrad or high level of tetrad counts, presenting a bimodal pattern. Further sequence comparison reveals substantial prokaryotic WD40 proteins (~20%) and a small part of eukaryotic WD40s (~2.0%, mainly from Fungi and Protista) are of high internal sequence identity, suggesting they should be formed from relatively young repeat duplication events in evolution. HR WD40s, most of which are very young, possess higher density of tetrads, and are thus presumably more stable. Our work will provide a basis for more in-depth investigations, such as studying the functions of certain prokaryotic WD40s, exploring the possible relationship between stability and evolvability by using HR WD40s as model systems, and so on.

## Methods

### Identification of WD40 proteins and abundance comparison

The UniProt Knowledgebase (UniProtKB, Release 2013_10)^23^ was used as input for the identification of WD40 proteins in this work. All the sequences and annotation data, including 45,288,084 entries (541,561 in Swiss-Prot and 44,746,523 in TrEMBL), were downloaded from ftp://ftp.uniprot.org.

A carefully designed pipeline was applied to identify and to curate putative WD40 proteins (Supplementary Fig. S2)^21^. In brief, to save computing resources, all the proteins were first coarsely pre-screened for a putative tetrad hydrogen bond network or a β-bulge, the two unique structural features in WD40 proteins^53^. Concerning that these criteria may have been too strict, we also included all the other potential WD40 proteins supported by the annotations of SMART^54^, Pfam^55^, PROSITE^56^, InterPro^57^, SUPFAM^58^, and Gene3D^59^. The protein sequences that passed the pre-screening were submitted to the WDSP program, which predicted the locations of each repeat, the hydrogen bond network positions, and the confidence scores of the predictions were also provided. The proteins whose repeat numbers are at least 6 and average repeat scores are no less than 48, suggested by the methodology of WDSP^21^, were retained. For the sake of reliability, the putative WD40 proteins whose scores are between 48 and 60 were double-checked based on annotations of SMART^54^, Pfam^55^, PROSITE^56^, and InterPro^57^. In order to remove redundancies, only the longest sequence was retained if one gene corresponded to multiple proteins in the same organism. This rule was actually derived from one of the curation criteria of the Swiss-Prot database.

For each entry in UniprotKB, the annotations in the fields of “OS” and “OC”, providing the source organism name and the taxonomic hierarchy, were parsed in order to inspect the taxonomic relationship. All the organisms were categorized into eight groups, including four eukaryotic groups (Animalia, Plantae, Fungi, and Protista), two prokaryotic groups (Archae and Bacteria), virus, and others.

An organism with a fully sequenced genome is supposed to have all its protein sequences deposited in the UniProtKB, and is assumed to have a complete proteome, as described in http://www.uniprot.org/keywords/KW-0181. The lists of proteins from complete proteomes were obtained from the UniProt website. For each of the proteomes, we removed the redundant proteins according to the rule described above. We then calculated the percentage of WD40 proteins in each proteome to measure the abundance. The one-sided Wilcoxon rank sum test was applied to evaluate the statistical significance of the WD40 abundance differences between different taxonomic categories, and the fold change was defined by the ratio of average WD40 percentages between the two taxonomic groups that were compared. As we lacked the knowledge of their distributions, it was reasonable to use nonparametric statistical tests.

### Construction of bacterial taxonomic relationships

The taxonomic relationships of the bacteria with complete proteomes were illustrated as a tree-like structure. Graphviz^60^, a graph layout and visualization package, was adopted to draw the diagram. In practice, we utilized PyGraphviz, an interface to Graphviz in the Python programing environment, to implement the illustration. In the constructed taxonomic tree, the radial lines from the inside to the outside denote taxonomic levels from phylum to species or even strain.

### Analysis of gene neighbourhood

We chose all the 7 Planctomycetes species that have WD40 genes for gene neighbourhood analysis. They are SINAD, ISOPI, PIRSD, PLAL2, PLABD, RHOBA, and PHYMF. As for Cyanobacteria, we selected the same number of species with high WD40 abundance from representative taxonomic groups. They are NOSP7, NOSS7, ANAVT, ANACC, CYAP2, ARTPN, and GLOVI. Their full names and taxonomic lineages were extracted from the UniprotKB (Release 2013_10), and are listed in Supplementary Table S3.

The annotations of these proteomes, including their gene locations in the genomes, transcription directions, and putative functions, were downloaded from http://bacteria.ensembl.org ^61^. The criteria of defining gene neighbourhood (or gene cluster) were derived from the literatures^30, 31^. In brief, the neighbouring genes should be in the same transcriptional direction, and the distance between adjacent genes should be less than 300 bp. After defining the gene neighbourhoods of all these WD40 proteins, the functional annotation phrases of these genes were collected to infer the WD40 genes’ functions. We removed phrases like “hypothetical protein”, “WD40 repeat”, *et al*., as they cannot provide useful information. The remaining phrases and their counts were adopted to draw word clouds using the package of WordCloud in the R statistical environment (https://cran.r-project.org/web/packages/wordcloud2/).

In order to find the gene neighbourhood conserved in different Cyanobacteria species, we first ran BLAST to establish the bidirectional best hit (BBH) relationships between WD40 proteins from a pair of species by sorting the bitscores and e-values^49^. This is a common strategy for identifying orthologs, such as in COG^62^. If the gene neighbours of a pair of BBH WD40 proteins further showed high sequence identities accordingly, they were recognized as a pair of conserved WD40 gene neighbourhoods (gene clusters). If a gene cluster in a lineage or species cannot find any detectable homolog from other lineages or species, it was treated as lineage-specific or species-specific.

### Generation of sequence logos

Conventionally, the four strands of a typical WD40 blade are labelled as S_a_, S_b_, S_c_, and S_d_, and the loops connecting them are named as L_ab_, L_bc_, L_cd_, and L_da_, respectively. Based on the secondary structures predicted by WDSP, all the four β-strands were aligned exactly by their positions. The loop regions were aligned by ClustalX using default parameters^63^. After aligning, the top 6, 5, 3, and 4 conserved sites were kept for L_da_, L_ab_, L_bc_, and L_cd_, respectively. An in-house Python script was used to draw the sequence logos.

### Internal sequence identity of WD40 proteins

The pairwise sequence alignment of repeats within the same WD40 protein were conducted by using the pairwise2 and MatrixInfo packages of bioPython^64^. The option of Needleman-Wunsch global alignment^65^ algorithm was chosen, and the sequence identity of an alignment was calculated by dividing the counts of identical residue pairs by the length of the shorter of the two sequences involved in the alignment.

We adopted the pairwise sequence identities between repeats to measure the level of internal sequence identity (or internal identity in short) of a protein. Within any eight contiguous repeats, if there exists at least six repeats whose pairwise alignment identities are all above a specified threshold X, we defined the internal sequence identity of this protein as at least X. If there were only six or seven repeats in a protein, the constraint of eight contiguous repeats was removed. The amounts of WD40 proteins at different levels of internal sequence identity in different taxonomic groups were represented as areas in an inverted triangle. We defined the proteins with internal sequence identity of at least 0.7 as Highly-Repetitive (HR) WD40 proteins, and the ones of at least 0.4, but excluding HR, as Moderately-Repetitive (MR).

### Analysis of synonymous substitution rate (*d*S) and ω

Based on the outputs of WDSP program^21^, we curated the multiple sequence alignments between WD40 repeats within each HR WD40 by visual inspection and manual adjustment. We then retrieved the corresponding DNA sequences for the HR WD40s from the ENA database^66^, and built the DNA alignment accordingly. Based on these DNA alignments, we used PAML to estimate the *d*S for each pair of repeat units within the same protein by using maximum-likelihood methods^41, 42^. In order to estimate the *d*S values for bacterial genes more precisely, we modified the source codes of PAML to incorporate the bacterial genetic code table, and recompiled them for this study. Those proteins with obsoleted DNA sequences and *d*S calculation with NAN error were not included in the next step. The median *d*S was chosen for each HR WD40. We finally obtained median *d*S values for 283 HR WD40s: 30, 113, and 140 WD40 proteins with the internal sequence identity level between 0.9–1.0, 0.8-0.9, and 0.7–0.8, respectively. For those HR WD40s with *d*S no less than 1.0, we further analysed the ω (the ratio of non-synonymous substitution rate to synonymous substitution rate) values estimated by PAML.

### Data availability

The datasets generated and/or analysed during the current study are available from the corresponding authors on reasonable request.

## Electronic supplementary material

Supplementary Information

Supplementary Dataset 1
