# Supplementary material for: Prokaryotic and Highly-Repetitive WD40 Proteins: A Systematic Study
Source: Sci Rep. 2017 Sep 6;7:10585. doi: 10.1038/s41598-017-11115-1 (PMC5587647; doi:10.1038/s41598-017-11115-1)
Supplement: Supplementary file 1 — Supplementary Information [file 41598_2017_11115_MOESM1_ESM.pdf]

Supplementary Information for "Prokaryotic and Highly-Repetitive WD40 Proteins: A Systematic Study"

Xue-Jia Hu[1,#], Tuan Li[1,#], Yang Wang[1], Yao Xiong[1], Xian-Hui Wu[1], De-Lin Zhang[1], Zhi-Qiang Ye[1,*] & Yun-Dong Wu[1,2,*]

[1] Lab of Computational Chemistry and Drug Design, Laboratory of Chemical Genomics, Peking University Shenzhen Graduate School, Shenzhen 518055, P. R. China

[2] College of Chemistry, Peking University, Beijing 100871, P. R. China

[#] These two authors contributed equally to this work.

[*] Correspondence and requests for materials should be addressed to Z.Q.Y. (email: yezq@pkusz.edu.cn) or Y.D.W. (email: wuyd@pkusz.edu.cn)

Supplementary Figures

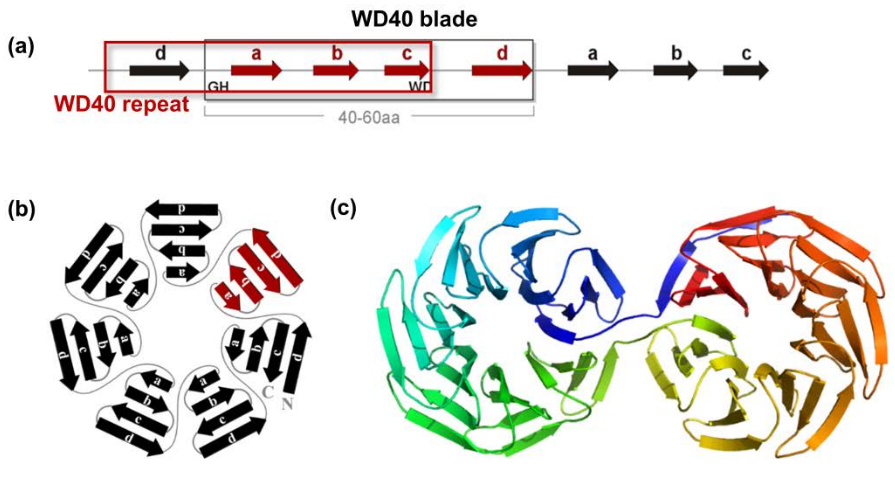

Supplementary Figure S1. Sequence and structure illustrations of WD40 domains.

(a) Schematic diagram of sequence repeat and structure blade. The strands are labelled with "a", "b", "c", and "d", and the conserved "GH" and "WD" motifs are shown. **(b)** Schematic diagram of a WD40 domain. **(c)** Cartoon presentation of a two-domain WD40 protein (Uniprot ID: B2J0I0_NOSP7; PDB ID: 2YMU; Gene Symbol: *Npun_R6612*).

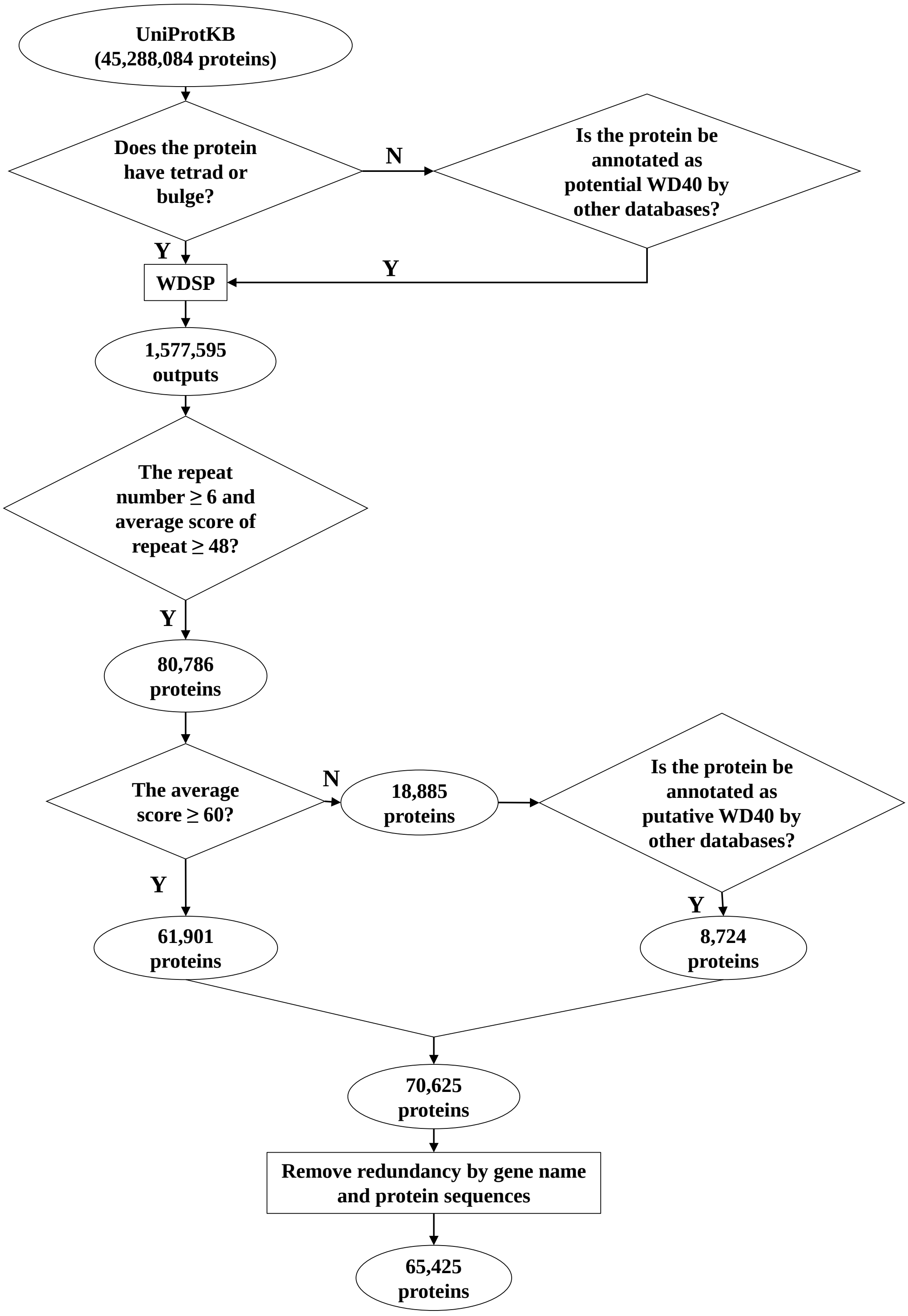

Supplementary Figure S2. Pipeline of WD40 protein identification and annotation.

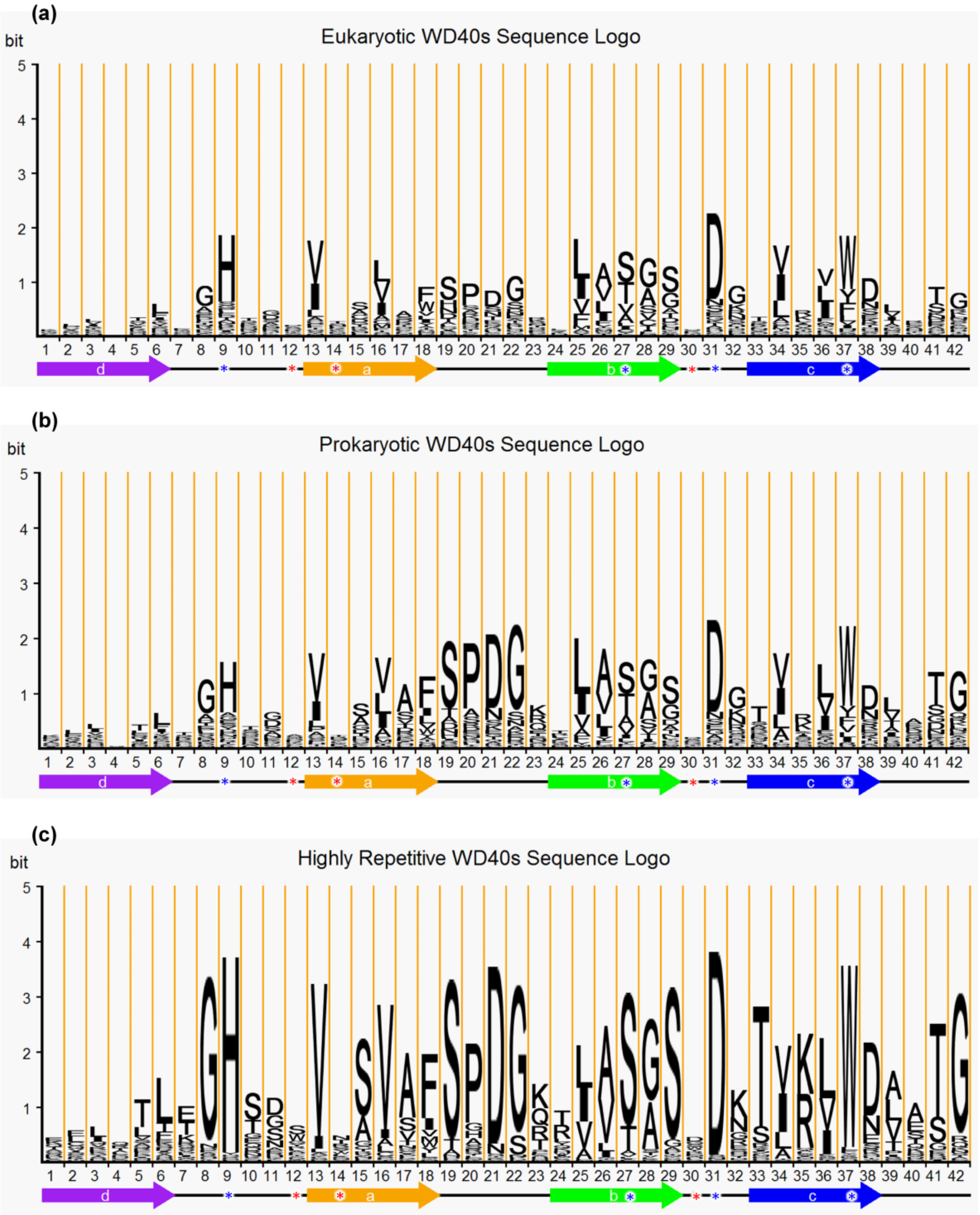

Supplementary Figure S3. Sequence logos of WD40 repeats from eukaryotic, prokaryotic, and Highly-Repetitive WD40 proteins.

The secondary structures are shown under the horizontal axis. The sites involved in tetrad hydrogen bond network are marked with blue stars, and the potential interaction hot spot sites are marked with red stars.

Repeats	score	Strand_d	Loop_da	Strand_a	Loop_ab	Strand_b	Loop_bc	Strand_c	Loop_cd	H_bonds
WD1	154	KERNRL	EAHSSS	VRGVAF	SPDGQ	TIASAS	DDK	TVKLWN	RNG	pentad
WD2	159	QLLQTL	TGHSSS	VWGVAF	SPDGQ	TIASAS	DDK	TVKLWN	RNG	pentad
WD3	158	QLLQTL	TGHSSS	VRGVAF	SPDGQ	TIASAS	DDK	TVKLWN	RNG	pentad
WD4	159	QLLQTL	TGHSSS	VWGVAF	SPDGQ	TIASAS	DDK	TVKLWN	RNG	pentad
WD5	159	QLLQTL	TGHSSS	VWGVAF	SPDGQ	TIASAS	DDK	TVKLWN	RNG	pentad
WD6	158	QLLQTL	TGHSSS	VRGVAF	SPDGQ	TIASAS	DDK	TVKLWN	RNG	pentad
WD7	158	QLLQTL	TGHSSS	VNGVAF	RPDGQ	TIASAS	DDK	TVKLWN	RNG	pentad
WD8	158	QLLQTL	TGHSSS	VWGVAF	SPDGQ	TIASAS	DDK	TVKLWN	RNG	pentad
WD9	153	QHLQTL	TGHSSS	VWGVAF	SPDGQ	TIASAS	DDK	TVKLWN	RNG	pentad
WD10	158	QLLQTL	TGHSSS	VRGVAF	SPDGQ	TIASAS	DDK	TVKLWN	RNG	pentad
WD11	158	QLLQTL	TGHSSS	VWGVAF	SPDDQ	TIASAS	DDK	TVKLWN	RNG	pentad
WD12	158	QLLQTL	TGHSSS	VRGVAF	SPDGQ	TIASAS	DDK	TVKLWN	RNG	pentad
WD13	159	QLLQTL	TGHSSS	VRGVAF	SPDGQ	TIASAS	DDK	TVKLWN	RNG	pentad
WD14	158	QLLQTL	TGHSSS	VWGVAF	SPDGQ	TIASAS	SDK	TVKLWN		pentad

Supplementary Figure S4. Repeat sequences of B2J0I0_NOSP7 (PDB ID: 2YMU; Gene Symbol: *Npun_R6612*).

Residues involved in the side chain hydrogen bond network are coloured in blue, and potential hotspot residues are coloured in red. The scores are outputs from the WDSP program.

Supplementary Tables

Supplementary Table S1. Overview of the number of WD40 proteins in bacterial phyla with complete proteomes available.

No.	Phylum	# of complete proteome	# of proteomes possessing WD40	# of WD40 proteins	# of WD40 proteins per proteome (average)
1	Planctomycetes	7	7	93	13
2	Cyanobacteria	50	41	399	10
3	Actinobacteria	182	58	248	4
4	Chloroflexi	16	8	32	4
5	Bacteroidetes	79	26	62	2
6	Chlorobi	11	11	22	2
7	Acidobacteria	8	6	12	2
8	Deferribacteres	4	2	4	2
9	Chlamydiae	30	4	7	2

10	Proteobacteria	766	265	413	2
11	Spirochaetes	44	4	6	2
12	Deinococcus-Thermus	16	7	10	1
13	Aquificae	9	5	6	1
14	Thermotogae	13	11	12	1
15	Firmicutes	364	5	5	1
16	Verrucomicrobia	4	2	2	1
17	Chrysiogenetes	1	1	1	1
18	candidate division WWE1	1	1	1	1
19	Fusobacteria	5	1	1	1
20	Thermobaculum	1	1	1	1
21	Tenericutes	50	0	0	n.a.
22	Synergistetes	4	0	0	n.a.
23	Nitrospirae	3	0	0	n.a.
24	Dictyoglomi	2	0	0	n.a.
25	Elusimicrobia	2	0	0	n.a.
26	Thermodesulfobacteria	2	0	0	n.a.
27	Ignavibacteriae	2	0	0	n.a.
28	Gemmatimonadetes	1	0	0	n.a.
29	Caldiserica	1	0	0	n.a.
30	Fibrobacteres	1	0	0	n.a.
	**Total**	**1679**	**466**	**1337**	**n.a.**

Supplementary Table S2. Top 10 bacterial organisms according to the rank of WD40 protein abundance.

No.	Organism	Phylum	# of WD40s	# of Proteins	WD40 percentage in proteome (%)
1	*Nostoc sp.* (strain ATCC 29411 / PCC 7524)	Cyanobacteria	27	5,330	0.51
2	*Acaryochloris marina* (strain MBIC 11017)	Cyanobacteria	40	7,897	0.51
3	*Anabaena variabilis* (strain ATCC 29413 / PCC 7937)	Cyanobacteria	28	5,591	0.50

4	*Singulisphaera acidiphila* (strain ATCC BAA-1392 / DSM 18658 / VKM B-2454 / MOB10)	Planctomycetes	35	7,062	0.50
5	*Nostoc punctiforme* (strain ATCC 29133 / PCC 73102)	Cyanobacteria	32	6,514	0.49
6	*Trichodesmium erythraeum* (strain IMS101)	Cyanobacteria	20	4,307	0.46
7	*Frankia sp.* (strain EuI1c)	Actinobacteria	30	7,032	0.43
8	*Arthrospira platensis* (strain NIES-39 / IAM M-135) (*Spirulina platensis*)	Cyanobacteria	24	5,865	0.41
9	*Nostoc sp.* (strain PCC 7120 / UTEX 2576)	Cyanobacteria	24	5,988	0.40
10	*Cyanothece sp.* (strain PCC 7822)	Cyanobacteria	24	6,498	0.37

Supplementary Table S3. The bacteria used in gene neighbourhood analysis.

Taxonomic Code	Full Name	# of WD40s	Taxonomic Lineage
NOSP7	*Nostoc punctiforme* (strain ATCC 29133/PCC 73102)	32	Cyanobacteria; Nostocales; Nostocaceae; *Nostoc*
NOSS7	*Nostoc sp.* (strain ATCC 29411/ PCC 7524)	27	Cyanobacteria; Nostocales; Nostocaceae; *Nostoc*
ANAVT	*Anabaena variabilis* (strain ATCC 29413/PCC 7937)	28	Cyanobacteria; Nostocales; Nostocaceae; *Anabaena*
ANACC	*Anabaena cylindrical* (strain ATCC 27899/PCC 7122)	20	Cyanobacteria; Nostocales; Nostocaceae; *Anabaena*
CYAP2	*Cyanothece sp.* (strain PCC 7822)	24	Cyanobacteria; Oscillatoriophycideae; Chroococcales; *Cyanothece*
ARTPN	*Arthrospira platensis* (strain NIES-39/IAM M-135) (*Spirulina platensis*)	24	Cyanobacteria; Oscillatoriophycideae; Oscillatoriales; *Arthrospira*
GLOVI	*Gloeobacter violaceus* (strain PCC 7421)	15	Cyanobacteria; Gloeobacteria; Gloeobacterales; *Gloeobacter*
SINAD	*Singulisphaera acidiphila* (strain ATCC BAA-1392/ DSM 18658/VKM B-2454/MOB10)	35	Planctomycetes; Planctomycetia; Planctomycetales; Planctomycetaceae; *Singulisphaera*

ISOPI	*Isosphaera pallida* (strain ATCC 43644/ DSM 9630/IS1B)	13	Planctomycetes; Planctomycetia; Planctomycetales; Planctomycetaceae; *Isosphaera*
PIRSD	*Pirellula staleyi* (strain ATCC 27377/DSM 6068/ ICPB 4128) (Pirella staleyi)	14	Planctomycetes; Planctomycetia; Planctomycetales; Planctomycetaceae; *Pirellula*
PLAL2	*Planctomyces limnophilus* (strain ATCC 43296/DSM 3776/ IFAM 1008/290)	11	Planctomycetes; Planctomycetia; Planctomycetales; Planctomycetaceae; *Planctomyces*
PLABD	*Planctomyces brasiliensis* (strain ATCC 49424/DSM 5305/ JCM 21570/NBRC 103401/ IFAM 1448)	8	Planctomycetes; Planctomycetia; Planctomycetales; Planctomycetaceae; *Planctomyces*
RHOBA	*Rhodopirellula baltica* (strain SH1)	11	Planctomycetes; Planctomycetia; Planctomycetales; Planctomycetaceae; *Rhodopirellula*
PHYMF	*Phycisphaera mikurensis* (strain NBRC 102666/KCTC 22515/ FYK2301M01)	1	Planctomycetes; Phycisphaerae; Phycisphaerales; Phycisphaeraceae; *Phycisphaera*

Supplementary Table S4. Details of the examples of the conserved and the lineage-specific WD40 gene neighbourhood.

Taxonomic Code	Gene Cluster 1	Sequence Identity (%) to NOSP7	Query Coverage (%)	Hit Coverage (%)	Gene Cluster 2
NOSP7	*Npun_R3841*, *Npun_R3840* (B2J567), *Npun_R3839*	-	-	-	*Npun_AF168* (B2JAS7), *Npun_AF169* (B2JAS8), *Npun_AF170*, *Npun_AF171*, *Npun_AF172*
NOSS7	*Nos7524_1785*, *Nos7524_1784* (K9QPU5), *Nos7524_1783*	90, 83, 54	100, 99, 99	100, 99, 99	-
ANAVT	*Ava_0285*, *Ava_0286* (Q3MGH4), *Ava_0287*	90, 85, 57	100, 99, 96	100, 96, 90	-
ANACC	*Anacy_1685*, *Anacy_1686* (K9ZFS1), *Anacy_1687*	89, 51, 79	100, 96, 99	100, 98, 98	-
GLOVI	*BAC88475*, *BAC88476* (Q7NN78), *BAC88477*	67, 32, -	70, 99, -	50, 99, -	-

Note: The gene cluster 1 consists of 3 genes, one of which is a WD40 gene with Uniprot Accession given in the parentheses. The corresponding protein products from NOSP7 were used as queries to align to corresponding proteins (hits) from NOSS7, ANAVT, ANACC, and GLOVI. The sequence identities, the percentages of query coverage, and the percentages of hit coverage are listed. The gene cluster 2 consists of 5 genes, two of which are WD40 genes with Uniprot Accessions given in the parentheses. This gene cluster is specific in NOSP7, and there is no alignment

information available.

Supplementary Table S5. The number of WD40 proteins at different levels of internal sequence identity and in different taxonomic categories.

Internal sequence identity		**≥0**	**≥0.1**	**≥0.2**	**≥0.3**	**≥0.4**	**≥0.5**	**≥0.6**	**≥0.7**	**≥0.8**	**≥0.9**
Eukaryotes	Animalia	22705	22552	6698	306	6	0	0	0	0	0
	Plantae	7913	7884	2722	50	8	3	1	0	0	0
	Fungi	21407	21301	7828	737	417	287	166	109	61	16
	Protista	9089	9018	3223	235	135	104	72	40	16	1
Archaea		63	63	51	13	8	6	5	3	0	0
Bacteria		4187	4181	3131	1451	818	443	255	153	73	15
Virus		58	58	21	0	0	0	0	0	0	0
Other		3	3	2	1	0	0	0	0	0	0
Total		65425	65060	23676	2793	1392	843	499	305	150	32

Supplementary Methods

Homology between prokaryotic WD40 and LECA WD40 proteins

The LECA (last eukaryotic common ancestor) described by Koonin E.V., *et al*[1] was used in this study. It was inferred that this LECA should contain 3413 proteins, which were represented by KOG or TWOG clusters. This list was downloaded from ftp://ftp.ncbi.nih.gov/pub/koonin/Ancestors/coelomate_tree/KOG0303.set1.lst and the corresponding KOG clusters were obtained from ftp://ftp.ncbi.nih.gov/pub/COG/KOG . Checking the annotations resulted in 93 KOG and 3 TWOG clusters belonging to WD40. By using the member sequences of each WD40 KOG or TWOG as queries, we ran the BLASTP[2] against the 4250 prokaryotic WD40 proteins' sequences with the E-value cut-off of 1E-4. If at least one member sequence of a KOG/TWOG could hit certain prokaryotic WD40 protein, we concluded that this LECA KOG/TWOG was homologous to some prokaryotic WD40 protein(s).

Analysis of LUCA WD40 proteins

Different research groups proposed different LUCA (last universal common ancestor) inferences. In this work, we tried two versions.

The first one was recently described by Weiss M. C., *et al* [3], and it was inferred that this LUCA should contain 355 genes, which was presented in the form of gene families and listed in the their supplementary materials. Most of these gene families were associated with PFAM annotations, and the PFAM ID of WD40 (PF00400) was checked. Further inspection was also performed for those several genes without PFAM annotations.

The second version of LUCA was presented in the database of eggNOG by Bork *et al* [4], and was integrated into UniProt Knowledgebase. The entries that could be mapped to LUCA were explicitly marked. We downloaded the entries of the 65425 WD40 proteins analysed in our work, and parsed the LUCA annotations for those having LUCA annotations accordingly.

Horizontal gene transfer (HGT) analysis of five species from Firmicutes

Our dataset contains only 5 WD40 proteins (J7IQH0_DESMD, C4Z165_EUBE2, D3E9J5_GEOS4, E6TQU9_BACCJ, and A8FAX4_BACP2) from 5 Firmicutes

species (DESMD, EUBE2, GEOS4, BACCJ, and BACP2), respectively. We adopted the HGT-Finder[5] to predict the potential horizontal transferred genes (HTG) for these 5 bacteria.

In detail, we first ran the sequence search tool DIAMOND[6] against the NR database from NCBI for each of the 5 proteomes. The parameters were adjusted to output the hits as many as possible, say, 1 million (the default is 25), in order to avoid missing hits with remote homology. Second, the sequence similarity search results were fed to HGT-Finder using a series of its parameters of R values (0.2, 0.3, 0.4, 0.5, 0.6, 0.7, 0.8, and 0.9), which could detect both ancient and recent HTGs. The results were combined to build up the list of candidate HTGs. By further filtering with Q-values less than 0.01, we can find potential HTGs with high likelihood. According to this setting, we found 89, 54, 73, 39, and 15 HTG candidates with high likelihood in DESMD, EUBE2, GEOS4, BACCJ, and BACP2, respectively. By checking whether these 5 WD40 proteins exist in these candidates, we can infer that whether they might be horizontal transferred from other species.

References

1 Koonin, E. V. *et al.* A comprehensive evolutionary classification of proteins encoded in complete eukaryotic genomes. *Genome Biol.* **5**, R7, doi:10.1186/gb-2004-5-2-r7 (2004).

2 Camacho, C. *et al.* BLAST+: architecture and applications. *BMC Bioinformatics* **10**, 421, doi:10.1186/1471-2105-10-421 (2009).

3 Weiss, M. C. *et al.* The physiology and habitat of the last universal common ancestor. *Nat Microbiol* **1**, 16116, doi:10.1038/nmicrobiol.2016.116 (2016).

4 Huerta-Cepas, J. *et al.* eggNOG 4.5: a hierarchical orthology framework with improved functional annotations for eukaryotic, prokaryotic and viral sequences. *Nucleic Acids Res.* **44**, D286-293, doi:10.1093/nar/gkv1248 (2016).

5 Nguyen, M., Ekstrom, A., Li, X. & Yin, Y. HGT-Finder: A New Tool for Horizontal Gene

Transfer Finding and Application to Aspergillus genomes. *Toxins* **7**, 4035-4053, doi:10.3390/toxins7104035 (2015).

6 Buchfink, B., Xie, C. & Huson, D. H. Fast and sensitive protein alignment using DIAMOND. *Nat. Meth.* **12**, 59-60, doi:10.1038/nmeth.3176 (2015).
